# Tropical rock lobster (*Panulirus ornatus*) uses chemoreception via the antennular lateral flagellum to identify conspecific ecdysis

**DOI:** 10.1038/s41598-023-39567-8

**Published:** 2023-07-31

**Authors:** Tara R. Kelly, Quinn P. Fitzgibbon, Gregory G. Smith, Thomas M. Banks, Tomer Ventura

**Affiliations:** 1 Institute for Marine and Antarctic Studies (IMAS), University of Tasmania, Private Bag 49, Hobart, TAS 7001 Australia; 2 Centre for Bioinnovation, School of Science, Technology and Engineering, University of the Sunshine Coast, 4 Locked Bag, Maroochydore, QLD 4558 Australia

**Keywords:** Computational biology and bioinformatics, Zoology

## Abstract

The tropical rock lobster, *Panulirus ornatus*, is a commercially important aquaculture species exhibiting complex social interactions in laboratory culture, including cannibalism of moulting conspecifics. Cannibalism of soft-shelled post-moult stage individuals is a major limitation during the juvenile stage of culture. Not limited to *P. ornatus*, cannibalism is widespread across farmed decapods, limiting stocking densities in crab, freshwater crayfish, and prawn species. To understand the mechanisms driving this behaviour and reduce its prevalence, we have investigated the role of chemoreception via the aesthetasc-bearing region of the lateral antennular flagellum, in the recognition of conspecific moulting cues. Differential expression analysis of several tissues in *P. ornatus* shows an upregulation of 70 ionotropic receptor isoforms, including co-receptors (IR25a and IR93a) and divergent receptors (IR4, IR7, and IR21a) in the aesthetasc-bearing region of the antennules. Deafferentation of the aesthetascs via deionised water exposure prevents juveniles from responding to conspecific moulting cues in a two-current choice flume, suggesting chemoreception, possibly olfaction, plays a role in identifying moulting juveniles. This is the first step in understanding the mechanisms via which cannibalism is triggered in juvenile *P. ornatus* culture. Further work in this area will help discover means to limit cannibalism in laboratory and commercial culture.

## Introduction

*Panulirus ornatus* is an emergent candidate for onshore commercial aquaculture due to its rapid growth and high value in the international market^1,2^. Substantial advances have been made to allow for closed system larval culture and current research aims to improve growth and survival of *P. ornatus* at the juvenile stage^3–5^. Survival rates at this stage are hindered by a high incidence of cannibalism in communal culture, where vulnerable, soft-shelled, post-moult individuals are attacked and cannibalised^5,6^. Isolated culture conditions remove the opportunity for cannibalism, however survival rates under isolated rearing are only slightly improved, and the trade-off is reduced growth rates, a vital factor in communal culture^5,7^. Additionally, isolated culture increases the potential cost of husbandry, which is unfeasible for large scale aquaculture, highlighting the importance of controlling cannibalism in this species to maintain optimal growth and health. Chemical communication is broadly used by crustaceans to interact with each other and their environment^8,9^. Chemical cues released by moulting crustaceans are recognised by conspecifics, indicating that chemoreception plays a key role in mediating cannibalism of post-moults^10,11^. Identifying the sensory pathways responsible for recognising moulting conspecifics is therefore essential to our understanding of cannibalistic behaviour in juvenile *P. ornatus.* Here we investigate the transcriptome of the chemoreceptor-rich aesthetasc-bearing region of the lobster lateral antennular flagellum and consider its role in detecting and mediating behavioural response to conspecific moulting cues.

With respect to intraspecific chemical cues, the olfactory pathway detects and mediates behavioural responses to conspecific courtship, alarm, aggregation, and social cues^11,12^. Chemoreceptors are prevalent across the spiny lobsters’ integument, however olfaction is uniquely facilitated by aesthetasc chemosensilla, located on the distal end of the lateral flagellum of the antennules, which are innervated with olfactory receptor neurons (ORNs)^9,13^. Olfactory receptor neurons are bipolar, meaning they have both an axon and dendrite extension, allowing the neuron to both transmit and receive sensory information. ORN axons innervate paired olfactory lobes in the midbrain, which consists of highly dense neuropil, allowing for multifaceted recognition of a wide range of olfactory cues^14^.

Ion channels, such as ionotropic receptors (IRs), are membrane bound receptor proteins, which when bound by a ligand, open an ion channel to allow ion flow into the cell. IRs have been found in the chemoreceptor organs of several crustaceans^9,15^ and are found in the lateral antennular flagellum, legs, and brain of the spiny lobster *Panulirus argus*^16^. They serve as the crustacean variant of the insect ionotropic glutamate receptors (iGluRs) which function in synaptic communication pathways throughout the nervous system. IRs function as combinations of subunits which are either broadly expressed or are species- or odorant-specific^16–19^. Co-receptor IRs (co-IRs) are required to form a functional receptor channel and are conserved across all protostomes studied to date, whilst the more recently evolved divergent IRs provide chemical specificity and are found to be conserved within phyla or species^17,19^.

In *P. argus*, several chemoreceptor proteins have increased expression in the brain, eye stalk, and hepatopancreas^16,20^, however research to date indicates IRs in spiny lobster ORNs mediate odorant input and olfactory signalling^20^. Apart from olfactory chemosensilla, the distal lateral flagellum of the antennules also contains mechanosensilla for hydrodynamic reception and bimodal contact chemo-mechanoreceptors^21^. Hydrodynamic-olfactory co-operation is important for odorant detection, as fluid turbulence encourages antennular flicking, which allows for improved odorant detection by increasing aesthetasc sensilla exposure to new water currents^22–24^. Ablation of antennular sensilla shows olfactory and non-olfactory receptor neurons have complementary functions allowing for food localisation via odorants^25,26^. Olfactory and distributed chemoreceptors on the antennules can detect some of the same food molecules and co-ordinate to drive searching behaviour^12,27^.

For olfactory inhibition, aesthetasc ablations, during which the animal is restrained and aesthetasc setae are shaved under a dissecting microscope, can be very complex, time consuming and a potential stressor for the early juveniles examined here^25,28,29^. Alternatively, exposure to deionised water has been proven to temporarily inhibit chemoreceptors in the aesthetasc-bearing region by interrupting the osmotic balance of chemoreceptors, including ORNs, thereby impeding olfaction^25,28,30^. Research such as this has already demonstrated the vital role of olfaction in localisation of food sources. To elucidate the pathways mediating cannibalistic behaviour in juvenile *P. ornatus,* we need to understand the role of chemoreception, such as olfaction, in recognising and responding to cues released by vulnerable moulting lobsters. Two-current choice flumes are well suited for the study of chemosensory behaviour as they allow animals to move between currents carrying different chemical cues with no physical divider in place^31^. This provides more information than other methods, such as Y-mazes and shuttle box systems, where results are often binary. Previous research has established two-current choice flumes to be a suitable tool to measure behavioural response in *P. ornatus* juveniles^10^. Kelly^10^ identified a preference response to conspecific moulting cues in familiar and naïve pairings, providing an effective behavioural assay, which is used here to assess the role of olfaction in detecting conspecific moulting cues.

This study aims to investigate the transcriptome of the aesthetasc-bearing region of *P. ornatus* lateral flagellum of the antennules, containing chemosensory neurons including ORNs, in addition to a functional ablation experiment. We compare the response of *P. ornatus* juveniles with either temporarily ablated (exposed to deionised water) or non-ablated distal antennules to conspecific moulting cues using a two-current choice flume assay. Understanding the role of chemical communication via the aesthetasc-bearing region of the antennules in *P. ornatus* recognition of moulting conspecifics, indicates the pathways responsible for mediating cannibalism of post-moult juveniles and may guide research to limit cannibalism in juvenile *P. ornatus* culture.

## Materials and methods

### Transcriptomic analysis

#### Sample preparation and sequencing

Multiple tissues were previously sampled and used for a transcriptomic assembly, published in Ventura, 2020^32^ (available in the NCBI BioProject: PRJNA903480). Raw reads from this project were included in the current study, from brain (n = 2), eye stalk (n = 2), antennal gland (n = 2), epithelium from beneath the dorsal carapace (n = 2) and hepatopancreas (n = 6) tissues. Adult *P. ornatus* were purchased from Torres Strait wild-caught stocks and reared at the University of the Sunshine Coast. Tissue from the aesthetasc-bearing region of the lateral flagellum of the antennules was sampled from inter-moult adult lobsters (male n = 1, female n = 3), snap-frozen in liquid nitrogen and stored at − 80 °C until further analysis. Frozen tissue was ground with mortar and pestle in liquid nitrogen and total RNA extracted with RNAzol (MRC) and β-Mercaptoethanol. Extracted RNA were assessed using NanoDrop 2000 (ThermoFisher) for yield and purity. For each sample a minimum 10 µl of clean RNA were combined with equal parts RNAstable LD (Sigma-Aldrich), then dried in Concentrator plus (Eppendorf) for 3 h at 60 °C. Desiccated RNA were sent to Novogene for quality control, library preparation, and RNA Sequencing (HiSeq2500, paired end 150 bp). RNA sequence data were submitted to the NCBI Short Read Archive under the BioProject: PRJNA903480 (BioSamples: SAMN32802504, SAMN32802505, SAMN32802506 and SAMN32802507).

#### Transcriptome assembly and alignment

The newly sequenced antennules, as well as the previously sequenced brain, eye stalk, antennal gland, epithelial tissue and hepatopancreas were used for transcriptomic analysis in this study. Raw paired-end reads for the antennule samples were quality checked using FASTQC^33^ and low quality bases (quality score < 20) were removed with Trimmomatic^34^. The trimmed reads were quality checked, concatenated, and de novo assembled alongside the previously sampled tissues with Trinity v.2.9.1^35^ as the draft reference genome and transcriptomes currently available for *P. ornatus* are preliminary and do not include the antennules. A total of 577,782,989 trimmed paired-end reads from all tissues were assembled, with a minimum contig length of 200 bp and a minimum K-mer count of 2. Transcript abundance of the 18 samples was then quantified using the trimmed paired end reads against the multiple tissue reference assembly with RSEM and Bowtie2^36,37^. BUSCO v3 was run on the de novo transcriptome assembly to assess its completeness using the *Arthropoda* lineage (number of genomes: 90, number of BUSCOs: 1013)^38^. Of the BUSCO groups, 98.12% have complete gene representation, while 1.18% are partially recovered and 0.69% are missing. The assembled transcriptome was screened with InterProScan (v.5.59-91.0)^39^ for conserved Pfam domains, PF00060 and PF10613, present in the ligand binding domain of IRs.

#### Bioinformatics analyses

The edgeR package in OmicsBox was used to conduct pairwise differential expression analysis comparing antennule tissue samples (n = 4) to non-antennule tissue samples (n = 14)^40,41^. Low counts were filtered to CPM1, normalised with TMM and the significance threshold set to 0.05. Transcripts from this analysis with a logFC > 1 were considered up-regulated and logFC < − 1 considered down-regulated. A Fisher’s exact test was conducted within the OmicsBox software to establish statistical significance between a test-set of differentially expressed genes and an annotated reference set. Transcripts classified as differentially expressed were then annotated within OmicsBox software using NCBI-BLAST + 2.10.0 nucleotide database with an Arthropoda taxonomy filter (e-values < 0.01)^42^. The resulting transcripts were further annotated using Gene Ontology (GO) mapping and a Gene Set Enrichment Analysis (GSEA) was performed on TMM normalised gene counts to identify enriched GO terms within the differentially expressed transcripts alongside all annotated features in the antennule transcriptomic assembly in OmicsBox^43^. Results were filtered to meet FDR < 0.05 significance threshold and a heatmap was generated using the OmicsBox software to visualise expression patterns of transcripts of interest which were differentially expressed in antennule tissue compared to non-antennule tissues (brain, eyestalk, hepatopancreas, antennal gland and epithelium tissue).

From this list of differentially expressed transcripts, 70 putative IRs were identified and open reading frames were predicted using OrfPredcitor v2.3^44^. A multiple sequence alignment was then performed with ClustalW^45^ including previously characterised IR25a sequences from *P. argus* and *Procambarus clarkii,* and IR93a and IR21a sequences from *H. americanus* sourced from NCBI. A maximum likelihood phylogenetic tree was produced with MEGA11^46^ with 1000 bootstrap replications using the JTT model. Alignments are provided in Supplementary File S1. The maximum likelihood tree was visualised using iTOL^47^.

### Olfactory response bioassay

#### Experimental animals

*Panulirus ornatus* individuals were reared from eggs at Ornatas commercial aquaculture facility following established procedures^48–50^. A total of 18 *P. ornatus* juveniles (instar J3, 1.63 g ± 0.1, carapace length 11.6 mm  ± 0.2) were stocked in three groups of six in 18 L opaque plastic tanks containing PVC tube hides lined with fly mesh for shelter. Water quality was monitored daily and maintained at an average temperature of 27.1 °C ± 0.1, dissolved oxygen 100.9% ± 0.3, salinity 33.9 ppt ± 0.07, and pH 8.2 ± 0.01. Lobsters were fed IMAS commercial-in-confidence feed, rationed at 5% of total tank biomass per 24 h, over six feeds.

Tanks were syphoned twice daily to remove uneaten feed and waste. Housing tanks were kept under a 12:12 L:D photoperiod, with purple (70% red, 30% blue) LED light (1950 lm) positioned 2.5 m above tanks to provide day phase. Lobsters were assigned an ID number and tagged with waterproof paper, glued to the dorsal carapace using gel super glue. At stocking the sex and wet weight (g) of juveniles was recorded, and wet weight was re-recorded the day following ecdysis throughout the experimental period. Pre-moult lobsters were identified up to 18 h prior to ecdysis by the darkening of their ecdysial suture line, on the dorsal and anterior edges of the gill cover (Supplementary Fig. S1).

#### Experimental protocol

**Figure 1:**
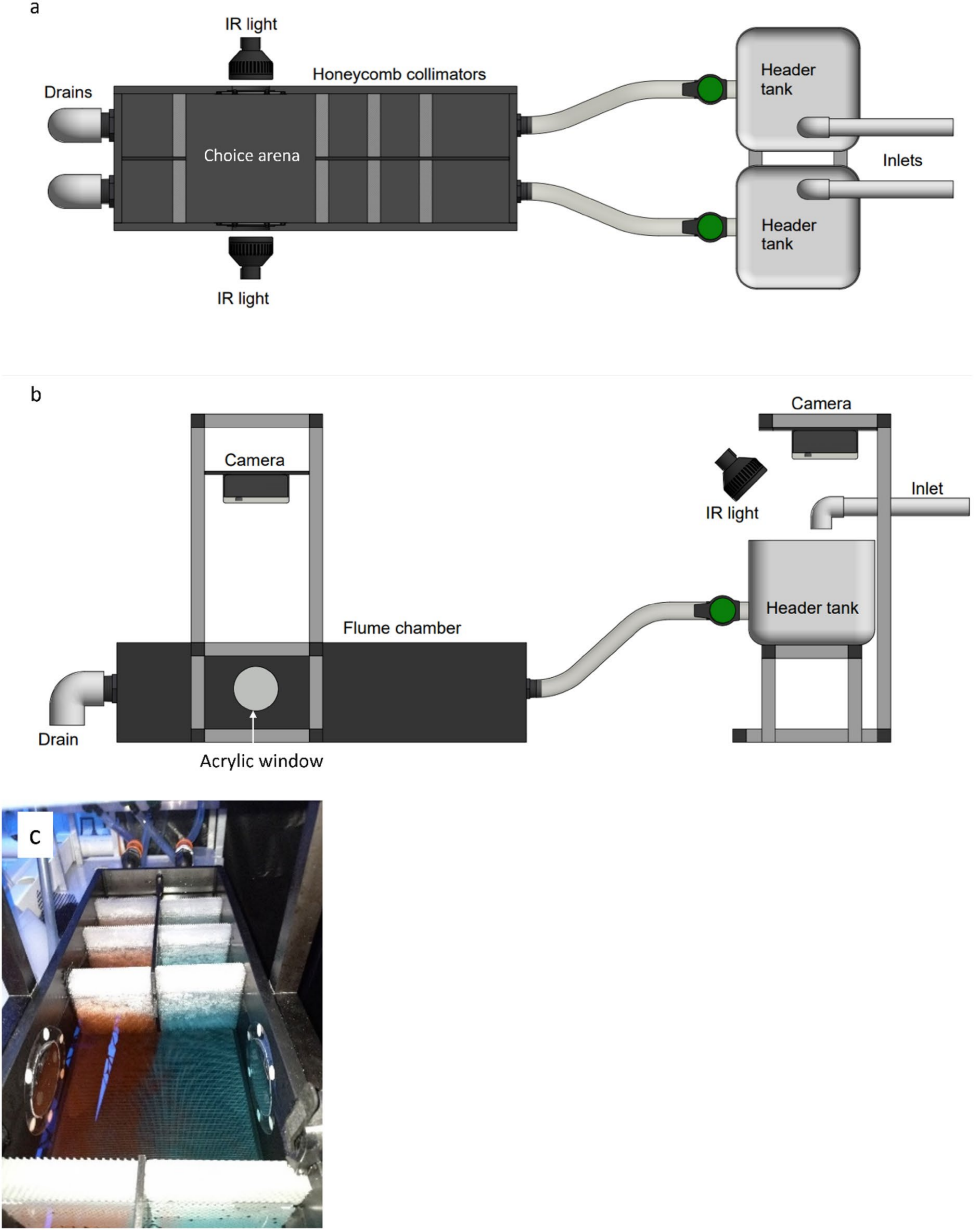
(**a**) Top-down schematic of two-current choice flume used for this study. Water enters both header tanks at 2 L min^−1^, then flows into main flume chamber, passing through three layers of honeycomb collimators to produce two distinct laminar currents in the choice arena. The choice arena is illuminated by two infrared lights placed at clear acrylic windows. (**b**) Side view schematic. Cameras in waterproof housing are secured to support stands and located directly above the header tanks and the choice arena. The header tanks are illuminated by a single infrared light placed at an angle to minimise surface reflection. (**c**) Demonstration of laminar flow in choice arena using red and blue dye, flow rate of 2 L min^−1^. Figure previously published by Kelly^10^.

Design of the two-current choice flume was based on recommendations by Jutfelt, 2017^31^ and detailed in Kelly^10^ (Fig. 1a,b). Water was supplied to two header tanks (2 L min^−1^ each) upstream of the flume chamber. Three layers of honeycomb collimators created two laminar currents in the choice arena. Laminar flow was confirmed by adding dye to each header tank and observing no mixing of currents within the choice arena (Fig. 1c). All tank elements were thoroughly cleaned between experimental runs. Removable pieces, such as honeycomb collimators and plastic tubing, were submerged in a chlorine bath (sodium hypochlorite 50 ppm) for 4 h, then rinsed under a flow of saltwater. The flume and header tanks were drained, rinsed with diluted chlorine, and flushed with saltwater. Water quality in the flume tank was monitored before and after all experiments and maintained at an average temperature of 27.2 °C ± 0.1, dissolved oxygen 101.8% ± 0.2 salinity 33.7 ppt ± 0.09, and pH 8.2 ± 0.02.

The experiment followed the procedures of Kelly^10^ in which a pre-moult juvenile is placed in a randomly assigned flume header tank and an inter-moult juvenile who is socially naïve to the pre-moult lobster is placed in the choice arena. Time-lapse footage (1 fps) of both the header tank and choice arena is captured overnight to identify the exact time lobsters moult in the header tank. To examine the impact of olfactory deafferentation on *P. ornatus* behavioural response to conspecific moulting cues, temporary aesthetasc ablation of inter-moult lobsters was achieved by submerging the distal lateral antennule flagellum, featuring the aesthetasc, in distilled water for 30 s. Choice arena lobsters in non-ablation treatments and control replicates had their aesthetascs submerged in saltwater for 30 s. This process was carried out 3 h prior to lobster placement in choice arena. To limit handling and air exposure stress, lobsters were transferred to the header tank and choice arena in jugs of water (2 L) 1 h before recording began to allow for acclimatisation. Three experimental groups were used: ablation treatments (n = 6) and non-ablation treatments (n = 5) featuring an inter-moult lobster in the choice arena and a pre-moult lobster in one header tank, and experimental controls (n = 3) featuring an inter-moult lobster in the choice arena with no lobster in either header tank (Table 1).

**Table 1:** Details of two-current choice flume experimental groups.

Group	Header tank lobster moult stage	Choice arena lobster moult stage	Choice arena lobster aesthetasc condition	Social relationship	n
Ablation treatment	Pre-moult	Inter-moult	Ablated	Naïve	6
Non-ablation treatment	Pre-moult	Inter-moult	Intact	Naïve	5
Experimental control	None	Inter-moult	Intact	None	3

n = number of replicates per treatment.

#### Tracking

Time-lapse images were written to AVI format on MATLAB R2020b, with a frame rate of 6 fps. The ImageJ plugin, AnimalTracker, was used to track the activity of lobsters within the two laminar currents, which were designated as regions of interest using AnimalTracker Zone Designer^51,52^. Each frame was filtered using Animal Tracker’s inbuilt background subtraction and thresholding, then postprocessed to remove excess noise. A single reference point, the centroid of the detected lobster, was followed by AnimalTracker without concern for body orientation. Tracks were manually edited if tracker lost the reference point on the body for several frames. A baseline observation hour was analysed from 1 h after lobsters were placed in the flume. Movement of choice arena lobsters in treatment replicates was tracked for 1 h before and 1 h after the lobster in the header tank moulted. Lobsters in the choice arena during control replicates were tracked for 1 h before and 1 h after the mean moult time observed in treatment replicates, approximately 3.5 h after commencement of dark phase.

#### Statistical analyses

Data were assessed using non-parametric tests in R^53^ due to restricted sample size. Water current preference of lobsters in the choice arena was assessed with a Mann–Whitney U test comparing mean time spent in the conspecific moulting cue during the 10 min after an upstream moult for ablation and non-ablation treatments. Time spent in a single current was also assessed for the baseline hour and 1 h before upstream moult using a Kruskal–Wallis test for ablation and non-ablation treatments and control replicates.

## Results

### Transcriptomic analysis

#### De novo assembly of transcriptome

Eighteen libraries containing a total of 577,782,989 paired-end reads were fed into Trinity^35^ to produce a multiple-tissue reference assembly. Trinity produced a total of 896,602 transcripts with an average length of 720 bp (min. 177 bp, max. 29,641 bp), predicting 681,338 genes with an average of 1.316 isoforms (min. 1, max. 72, predicted 118,112 genes in aesthetasc-bearing antennule tissue). Using InterProScan to screen for the conserved Pfam domains, PF00060 and PF10613, identified 726 sequences for PF00060, and 345 sequences for PF10613 (Supplementary File S2 and S3 respectively).

#### Differential expression analysis

From 120,812 filtered features, 3205 were significantly differentially expressed (FDR < 0.05).

**Figure 2:**
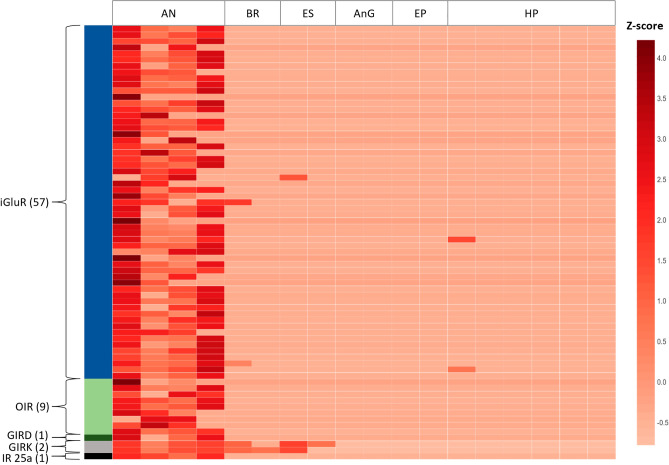
Heatmap of 70 differentially expressed transcripts (FDR < 0.05, logfold-change > 1) from six tissue types in adult *Panulirus ornatus.* Expression levels are shown as z-scores, represented by the colour key. *AN* antennule (n = 4), *BR* brain (n = 2), *ES* eye stalk (n = 2), *AnG* antennal gland (n = 2), *EP* epithelium (n = 2), *HP* hepatopancreas (n = 6). Upregulated transcripts align with several ionotropic receptor subunits. Fifty seven transcripts share identity with ionotropic glutamate receptor isoforms (iGluR, dark blue), nine transcripts share identity with olfactory ionotropic receptors (OIR, light green), one transcript shares identity with glutamate ionotropic receptor delta-like (GIRD, dark green), two transctripts share identity with glutamate ionotropic receptor kainate-like (GIRK, grey), and one transcript shares identity with ionotropic receptor IR25a (IR 25a, black).

**Figure 3:**
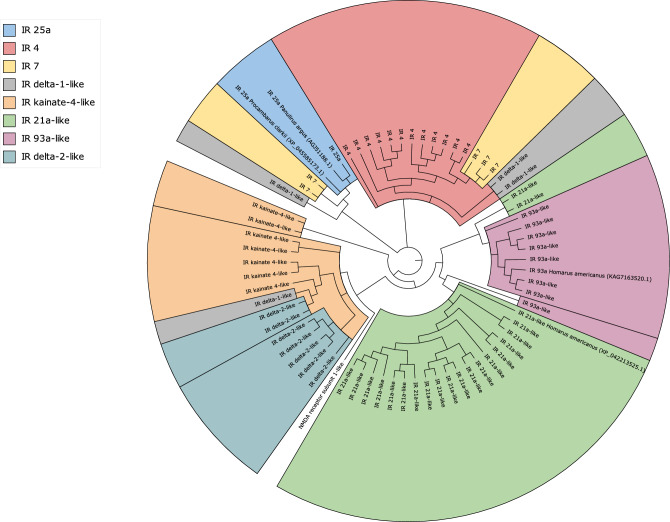
Maximum likelihood phylogenetic tree of IRs differentially expressed in inter-moult, adult, *P. ornatus* tissue from the aesthetasc-bearing region of the lateral antennular flagellum alongside IR homologues from *Panulirus argus, Procambarus clarkii* and *Homarus americanus* from NCBI database. Tree was built using MEGA11^46^ with 1000 Bootstrap replications under the JTT substitution model then visualised on iTOL v 6.7.3^47^.

This included, 2481 upregulated transcripts (logFC > 1), and 724 downregulated transcripts (logFC < − 1) in the aesthetasc-bearing antennule tissue compared to other tissues. NCBI nucleotide BLAST found successful hits for 1450 sequences (Supplementary File S4) and 70 ionotropic receptor transcripts were identified with upregulated expression in antennule tissue (Fig. 2). Annotation via the NCBI-BLAST + 2.10.0 nucleotide database with an Arthropoda taxonomy filter found 14 transcripts shared high identity with putative genes, while the remaining 56 share high identify with predicted homologues of ionotropic glutamate receptors. We have identified homologues for kainate-like receptors, delta-like receptors, two co-IRs (IR25a and IR93a), several divergent IRs (IR4, IR7 and IR21a) and one NMDA receptor (Fig. 3). IR25a was consistently upregulated in the aesthetasc-bearing region of the antennule and downregulated in all other tissues (Fig. 2). IR25a has been well described as a co-receptor expressed in *P. argus* olfactory receptor neurons (ORNs), and the sequence determined here in *P. ornatus* shares high identity with *P. argus* (92.49% ident., e-value 0.0, Supplementary File S4). Single cell transcriptome research has previously identified co-IRs, IR25a and IR93a in *P. argus* ORNs^54^. Our analysis similarly revealed an upregulation of these two co-IRs in the *P. ornatus* antennule tissue but failed to observe increased expression of the non-olfactory antennular co-IRs, IR8a and IR76b.

#### Enrichment analysis of Gene Ontology terms in antennule tissue

Functional gene set enrichment analysis (GSEA) of six tissues highlighted 78 Gene Ontology (GO) IDs, with 16 over-represented and 63 under-represented (FDR < 0.05). This provides mechanistic and functional insights into differentially expressed genes in the aesthetasc-bearing region of *P. ornatus* lateral antennular flagellum.

Forty-three Biological Process GO IDs were observed, with five over-represented (electron transport chain, cellular respiration, energy derivation by oxidation of organic compounds, Golgi vesicle transport, aerobic respiration), and 38 under-represented with several related to negative regulation of metabolic processes (Supplementary Table S1). Nineteen Cellular Component GO IDs were seen with four over-represented (Golgi apparatus, organelle sub compartment, respirasome, Golgi apparatus sub compartment) and 15 under-represented (Supplementary Table S2). Additionally, 16 Molecular Function GO IDs were enriched, seven over-represented (three involving receptor activity, three with gated ion channel activity and one electron transfer activity) and nine under-represented (Supplementary Table S1).

Three GO terms that were significantly over-represented and are of interest for this study are neurotransmitter receptor activity (GO:0030594), ionotropic glutamate receptor activity (GO:0004970) and glutamate receptor activity (GO:0008066). A set of 12 transcripts contribute directly to the enrichment of these GO terms in aesthetasc-bearing antennule tissue (Table 2). Several of these sequences have high identity to two genes characterised in *P. argus,* IR4 (KC603903.1) and IR7 (KC603904.1). Others have high sequence identity with various ionotropic glutamate receptors.

**Table 2:** Summary of BLAST hits for 12 transcripts contributing to over-representation of GO:0030594 neurotransmitter receptor activity, GO:0004970 ionotropic glutamate receptor activity and GO:0008066 glutamate receptor activity in the aesthetasc-bearing antennule region of adult *Panulirus ornatus*.

BLAST hit	Transcripts	Accession no	Identity
*Panulirus argus* olfactory ionotropic receptor IR7 mRNA, complete cds	2	KC603904.1	79%, 67%
*Panulirus argus* olfactory ionotropic receptor IR4 mRNA, complete cds	5	KC603903.1	95%, 81%, 81%, 78%, 79%
PREDICTED: *Litopenaeus vannamei* glutamate receptor ionotropic, kainate 2-like (LOC113813085), mRNA	1	XM_027365024.1	69%
PREDICTED: *Penaeus monodon* glutamate receptor ionotropic, delta-1-like (LOC119579633), mRNA	1	XM_037927547.1	69%
PREDICTED: *Litopenaeus vannamei* probable glutamate receptor (LOC113803339), mRNA	1	XM_027354110.1	76%
PREDICTED: *Penaeus monodon* ionotropic receptor 21a-like (LOC119598337), mRNA	1	XM_037947992.1	74%
PREDICTED: *Penaeus monodon* probable glutamate receptor (LOC119596669), mRNA	1	XM_037946000.1	66%

### Olfactory response bioassay

#### Flume current preference

**Figure 4:**
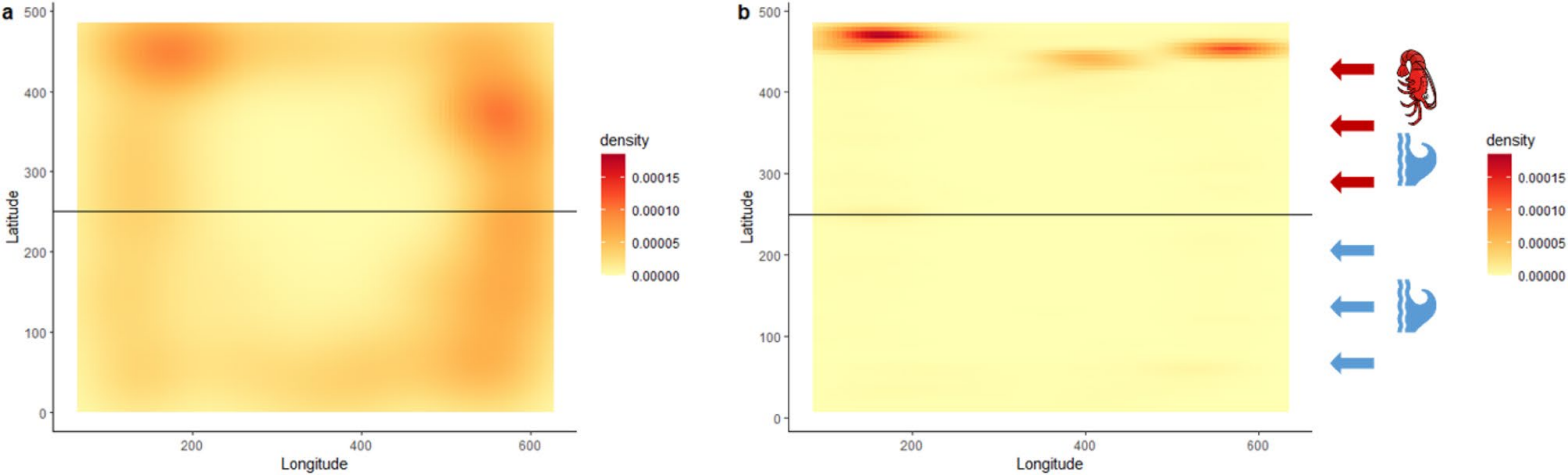
Density plot for number of detection points of lobster coordinate position in choice arena during 10 min exposure to conspecific moulting cues. (**a**) Inter-moult juveniles with ablated aesthetascs (n = 6). (**b**) Inter-moult juveniles with functional, non-ablated aesthetascs (n = 5). In each plot conspecific cue current is present above the horizontal line and water only current is present below the line. Water flow is from right to left.

Average moulting time of lobsters in the header tank was 3.65 h ± 0.4 after commencement of dark phase. This average was used as a pseudo-moult time when tracking movement and behaviour in control replicates. The water current preference of all choice arena lobsters was observed for a baseline hour showing no difference in the average time spent in a single current between lobsters with non-ablated or ablated aesthetascs, or control replicates (Table 3, Kruskal–Wallis test, H(2) = 0.8, *P* = 0.7). Water current preference during the 1 h before upstream lobsters moult (1 h before mean moult time for control replicates) was statistically similar between treatments and controls (Kruskal–Wallis test, H(2) = 2.9, *P* = 0.2), although the average time of non-ablated treatments is increased compared to the baseline (Table 3). Lobsters with non-ablated aesthetascs spend a greater amount of time in the moulting cue current than lobsters with ablated aesthetascs during the 10 min after a lobster moults upstream (Table 3, Mann–Whitney U test, U = 4, *P* = 0.04). This difference indicates the aesthetasc facilitates a preference response to conspecific moulting cues, which is visualised by a density plot mapping the coordinate points of lobsters within the choice arena during the 10 min moult cue exposure period (Fig. 4).

**Table 3:** Mean percentage time lobsters spent in the conspecific conditioned current of the choice arena for two treatments and a control group.

	n	Baseline hour	1 h before moult	10 min moult cue exposure
Ablation	6	63.7 ± 8	64.1 ± 8	64.6 ± 6
Non-ablation	5	66.4 ± 15	87.1 ± 2	91.0 ± 3*
Control	3	48.2 ± 10	64.0 ± 12	
Experiment total	14	59.4 ± 8	71.7 ± 5	

No upstream moults occurred in control treatments. Contol replicates were analysed for 1 h before mean moult time. Mean values ± s.e.m. n = number of replicates per treatment. *Denotes statistically significant value.

## Discussion

Investigation of the *P. ornatus* transcriptome has revealed significant upregulation of 70 ionotropic receptor transcripts within the aesthetasc-bearing region of the lateral antennular flagellum. Additionally, deafferentation of chemoreceptors in this region of the antennules prevents *P. ornatus* juveniles from responding to conspecific moulting cues. This demonstrates that chemoreception via the distal region of the antennular lateral flagellum is required to mediate a behavioural response to conspecific moulting cues. The aesthetasc setae in this region of the antennules are responsible for olfaction in spiny lobsters^9,13,27^, therefore we propose olfaction plays at least a partial role in recognition of conspecific moult cues. This finding is critical for research aimed to minimise cannibalism of vulnerable, soft-shelled, post-moult lobsters to improve juvenile culture productivity for this commercially important species.

Within the chemical rich environment of marine crustaceans, chemical communication is vital in facilitating many social behaviours, such as aggregation^28^, identifying familiar and naïve conspecifics^55^, differentiating between dominant and subordinate individuals^56,57^, as well as between inter-moult and moulting lobsters^10,58^. Spiny lobsters feature sensory sensilla over much of their body, particularly the antennules, mouthparts, and dactyls, innervated with both chemoreceptor and mechanoreceptor neurons, however, olfactory receptor neurons are only present in aesthetasc chemosensilla^9,13,27^. Chemical sensory pathways are responsible for receiving several forms of non-contact conspecific communication, such as courtship pheromones^12^, blood-borne injury cues^29^, and urine-borne cues responsible for mediating conspecific aggregation and aggressive behaviours^28,56^. Aiming to understand the mechanisms driving the detection, attack, and cannibalism of moulting and post-moult juveniles, we have examined the role of chemoreception via the lateral antennular flagellum, including the olfactory aesthetasc, in recognising conspecific moulting cues without physical or visual contact.

We can conclude from differential expression analysis and enrichment analysis that IR expression is enriched within the aesthetasc-bearing tuft of the antennules. Enrichment analysis found GO terms of key interest, neurotransmitter receptor activity, ionotropic receptor activity, and glutamate receptor activity, to be over-represented in the antennules. The genes associated with these GO terms have moderate to high sequence identity with several ionotropic receptors (IR) and ionotropic glutamate receptors (iGluR) in the Caribbean spiny lobster (*P. argus*), giant tiger prawn (*Penaeus monodon*) and the Pacific white shrimp (*Litopenaeus vannamei*). Phylogenetic conservation has been observed for various IRs with IR25a conserved in all protostomes studied to date^17,18^. The similarity in sequence, and more significantly, the phylogenetic similarity seen between the predicted co-IRs, 25a and 93a-like homologues, identified here in *P. ornatus* and confirmed homologues in *P. argus* and *H. americanus* supports our identification. Four co-IRs have been described in crustaceans, IR25a, IR93a, IR8a and IR76b. Single cell ORN transcriptomes produced by Kozma^54^, found *P. argus* ORNs only express two of the four co-IRs, IR25a and IR93a, the same two identified in the current study. IR25a and IR93a are obligate co-receptors expressed in essentially all ORNs^20^, and IR25a is also expressed in most CRNs of *P. argus* antennules, antennae, and dactyls^16,19^. The combination of obligate co-IRs with phyla conserved divergent IRs enables binding specificity for the receptor^17,19,20^. Divergent IRs, IR4, IR7 and IR21a-like, present upregulated expression in *P. ornatus* aesthetasc-bearing antennule tissue. Several transcripts for IR4 and IR7 contribute to the over-representation of key GO terms in the aesthetasc-bearing antennule tissue studied here.

In *P. argus*, both aesthetasc and non-aesthetasc chemoreceptor sensilla mediate lobster detection of, identification of, and orientation towards food sources^59^, however temporary deafferentation of aesthetasc chemoreceptor neurons does somewhat decrease success in finding food^25^. The use of dual antennular chemosensory pathways to mediating odorant activation for locating food in spiny lobsters enables a functional redundancy; if one type of chemoreceptor is inhibited, the other still fills the role. Our expression analysis demonstrates the significant presence of IRs in the lateral flagellum of the antennules of *P. ornatus,* however, to functionally annotate these receptors in the ORNs requires a cellular level loss-of-function investigation. This would be required to determine if aesthetasc sensilla alone detect conspecific moulting cues, and if so, present a new line of investigation to limit cannibalism in *P. ornatus* culture. Transcriptomic analysis of single ORN cells from *P. ornatus* aesthetascs, as previously done by Kozma^54^ for *P. argus,* would differentiate between IRs expressed in ORNs and CRNs in this region of the antennules. Functional assessment of IRs in chemosensory behaviour has been achieved through RNA interference (RNAi) knock-out of IR25a and IR93a in several *Daphnia* species^60^. In the case of *P.* *ornatus*, reverse genetic studies with RNAi have proven to be ineffective, due in part to animal-wide low expression of the enzymes and receptors required for functional and systemic gene silencing^61^. Despite multiple attempts to generate clear phenotypes with RNAi in *P.* *ornatus* and other Palinurid lobsters, no significant silencing has ever been observed which limits the functional annotation of ORNs in spiny lobsters.

Additionally, the development and functionality of chemosensory systems in crustaceans with drastically altered morphology across life-stages is understudied^12^, making it difficult to select appropriate targets for RNAi and develop assays to test for altered olfactory capacity. Continued research contributing to the transcriptomic database for *P. ornatus* across multiple life-stages may reveal a difference in IR expression patterns in the antennules, which may then inform on optimal genes to silence with RNAi when the technology becomes more accessible in *P. ornatus*.

With currently available knowledge we assessed the function of chemoreception via the aesthetasc-bearing region of the lateral antennular flagellum, in mediating a behavioural response to conspecific moulting cues. A two-current choice flume was used in a previous study with juvenile *P. ornatus* to identify preference for or against conspecific moulting cues, with a focus on the moult stage of the responding lobster and its relationship to the moulting lobster. This research found inter-moult lobsters are attracted to moulting cues from lobsters whom they are naïve to^10^. This bioassay was applied to the current study, pairing inter-moult lobsters with naïve, pre-moult lobsters, and tracking the time inter-moult lobsters spent in the conspecific cue current for 10 min following upstream ecdysis. Lobsters whose antennules were exposed to seawater only (non-ablated), responded as expected based on the aforementioned bioassay, spending an average 91.0 ± 3% of the 10 min in the moulting cue current. Alternatively, lobsters whose antennules were temporarily ablated with deionised water exposure spent an average 64.6 ± 6% of the 10 min in the moulting cue current, statistically similar to the baseline results for all treatment and control replicates. This indicates deafferentation of chemoreceptor neurons, including ORNs, in the aesthetasc-bearing region of the lateral antennular flagellum removes the ability to perceive a moulting conspecific via chemical cues alone. Flume assays have been highly effective in identifying preference behaviour in crustaceans, in response to various conspecific odours and feed attractants^58,62,63^. The current study is applicable to further aquaculture research and development, but also has great potential for fisheries and ecology as we need to understand how aquatic animals interact with each other and their rapidly changing habitats.

Cannibalism is one of the most prevalent limiting factors in the culture of several commercially significant species^1,6^, including *P. ornatus*^5^. Lobsters with temporarily ablated aesthetascs do not display preference for or against moulting cues, indicating they are incapable of responding to such cues without functional aesthetascs. The knowledge gained here indicates that antennule chemoreception, and possibly olfaction, are a link in cannibal-prey recognition. The identity of the chemical cue being received by cannibals remains a missing link in this research. Ecdysis is regulated by a multitude of hormones responsible for separating the external cuticle from the epidermis, and simultaneously forming a new cuticle. Numerous studies have indicated chemical cues released by moulting crustaceans influence conspecific behaviours associated with survival, aggression, and cannibalism^11,64^. An investigation into blood-borne and urine-borne cues, targeting molecules in high abundance at ecdysis, may elucidate the type of molecules responsible for indicating the biological state of lobsters at ecdysis to others.

## Conclusion

Here we endeavoured to uncover the chemosensory pathways responsible for receiving conspecific moulting cues and mediating *P. ornatus* response using transcriptomic analysis coupled with a functional bioassay. We successfully implemented the bioassay with functional ablation of the aesthetasc-bearing region of the lateral antennular flagellum, demonstrating the role of chemoreception in this region in detecting conspecific ecdysis. The accompanying transcriptomic analysis of this antennular region revealed upregulation of 70 ionotropic receptor transcripts, adding to the growing genomic and transcriptomic knowledge for this key aquaculture species. This study indicates that chemoreception via the aesthetasc-bearing region of the lateral antennular flagellum facilitates recognition of moulting conspecifics in the tropical rock lobster, *P. ornatus*. Furthermore, we propose this cue recognition plays a role in mediating cannibalism of post-moult juveniles in communal culture. Continued research in this area may aim to identify chemicals released into the environment at ecdysis and received by potential cannibals. As commercial culture for this species continues to develop, limiting cannibalism in communally cultured juveniles is essential to sustaining productivity.

## Supplementary Information

Supplementary Tables.

Supplementary Figures.

Supplementary Information 1.

Supplementary Information 2.

Supplementary Information 3.

Supplementary Information 4.

## Data Availability

RNA sequence data for this study has been submitted to the NCBI Sequence Read Archive (http://www.ncbi.nlm.nih.gov/sra under BioProject: PRJNA903480). All other data presented in this study is available on request from the corresponding author.
