# Supplementary material for: Tropical rock lobster (Panulirus ornatus) uses chemoreception via the antennular lateral flagellum to identify conspecific ecdysis
Source: Sci Rep. 2023 Jul 31;13:12409. doi: 10.1038/s41598-023-39567-8 (PMC10390513; doi:10.1038/s41598-023-39567-8)
Supplement: Supplementary file 1 — Supplementary Tables. [file 41598_2023_39567_MOESM1_ESM.pdf]

Supplementary Information

Tropical rock lobster (*Panulirus ornatus*) uses chemoreception via the antennular lateral flagellum to identify conspecific ecdysis

Tara Kelly[1*], Quinn Fitzgibbon[1], Gregory Smith[1], Thomas M. Banks[2], Tomer Ventura[2]

[1.]Institute for Marine and Antarctic Studies (IMAS), University of Tasmania, Private Bag 49, Hobart, TAS 7001, Australia.

[2.]Centre for Bioinnovation, School of Science, Technology and Engineering, University of the Sunshine Coast, 4 Locked Bag, Maroochydore, QLD 4558, Australia.

*Corresponding author: t.kelly@utas.edu.au

Supplementary File S1. FASTA sequences used to create Fig. 4.

Supplementary File S2. Pfam domain PF00060 sequences.

Supplementary File S3. Pfam domain PF10613 sequences.

Supplementary File S4. List of features significantly differentially expressed in tissue sampled from the distal end of the lateral antennule flagellum of adult *Panulirus ornatus.* Statistical values from differential expression analysis and results from NCBI nucleotide BLAST displayed.

Supplementary Fig S1. (a) Ecdysial suture line visible on gill cover of J3 instar *Panulirus ornatus*. Photo taken 12-18 h prior to ecdysis. (b) Inter-moult J3 instar *Panulirus ornatus*, no dark suture line visible.

Supplementary Table S1. Gene Set Enrichment Analysis- Biological Process GO terms over- and under-expressed in the aesthetasc-bearing region of the antennules in adult *Panulirus ornatus*.

Tags	GO ID	GO Name	ES	NES	FDR q-val	FWER p-val
OVER-EXPRESSED	GO:0022900	electron transport chain	0.483335	2.832604	0	0
OVER-EXPRESSED	GO:0045333	cellular respiration	0.419599	2.697469	8.62E-05	0.001
OVER-EXPRESSED	GO:0015980	energy derivation by oxidation of organic compounds	0.371649	2.466176	0.002349	0.035
OVER-EXPRESSED	GO:0048193	Golgi vesicle transport	0.391396	2.080324	0.027335	0.437
OVER-EXPRESSED	GO:0009060	aerobic respiration	0.389523	2.044016	0.031789	0.508
UNDER-EXPRESSED	GO:0043086	negative regulation of catalytic activity	-0.38873	-1.89776	0.045072	0.814
UNDER-EXPRESSED	GO:0000398	mRNA splicing, via spliceosome	-0.24297	-1.8925	0.045326	0.82
UNDER-EXPRESSED	GO:0000377	RNA splicing, via transesterification reactions with bulged adenosine as nucleophile	-0.24297	-1.89191	0.044595	0.821
UNDER-EXPRESSED	GO:0016311	dephosphorylation	-0.28301	-1.87879	0.04623	0.841
UNDER-EXPRESSED	GO:0019941	modification-dependent protein catabolic process	-0.25771	-1.85861	0.049373	0.867
UNDER-EXPRESSED	GO:0015931	nucleobase-containing compound transport	-0.3713	-1.84235	0.049859	0.881
UNDER-EXPRESSED	GO:0006325	chromatin organization	-0.41252	-3.39201	0	0
UNDER-EXPRESSED	GO:0071103	DNA conformation change	-0.54026	-2.93865	4.49E-04	0.001
UNDER-EXPRESSED	GO:0016570	histone modification	-0.4489	-2.83682	0.00138	0.004
UNDER-EXPRESSED	GO:0016569	covalent chromatin modification	-0.4489	-2.79716	0.001104	0.004
UNDER-EXPRESSED	GO:0009892	negative regulation of metabolic process	-0.32977	-2.6732	0.001182	0.007
UNDER-EXPRESSED	GO:0010605	negative regulation of macromolecule metabolic process	-0.32309	-2.60575	0.002213	0.015
UNDER-EXPRESSED	GO:0031324	negative regulation of cellular metabolic process	-0.37144	-2.45319	0.005495	0.05
UNDER-EXPRESSED	GO:0018205	peptidyl-lysine modification	-0.38548	-2.41737	0.006089	0.06
UNDER-EXPRESSED	GO:0008213	protein alkylation	-0.47973	-2.38864	0.006609	0.07
UNDER-EXPRESSED	GO:0006479	protein methylation	-0.47973	-2.37136	0.007148	0.08
UNDER-EXPRESSED	GO:0051172	negative regulation of nitrogen compound metabolic process	-0.38132	-2.35735	0.007291	0.086
UNDER-EXPRESSED	GO:0006357	regulation of transcription by RNA polymerase II	-0.28738	-2.3386	0.007496	0.099
UNDER-EXPRESSED	GO:0048731	system development	-0.34472	-2.28878	0.009518	0.14
UNDER-EXPRESSED	GO:0010629	negative regulation of gene expression	-0.37274	-2.24188	0.010507	0.181
UNDER-EXPRESSED	GO:0008380	RNA splicing	-0.26296	-2.18275	0.014325	0.251
UNDER-EXPRESSED	GO:0042254	ribosome biogenesis	-0.25255	-2.16241	0.015558	0.277
UNDER-EXPRESSED	GO:0006470	protein dephosphorylation	-0.35786	-2.15367	0.016076	0.296
UNDER-EXPRESSED	GO:0006366	transcription by RNA polymerase II	-0.23351	-2.14733	0.016118	0.303
UNDER-EXPRESSED	GO:0006397	mRNA processing	-0.21782	-2.14718	0.015599	0.303
UNDER-EXPRESSED	GO:0044257	cellular protein catabolic process	-0.26653	-2.08581	0.022231	0.414
UNDER-EXPRESSED	GO:0051603	proteolysis involved in cellular protein catabolic process	-0.26653	-2.08502	0.021694	0.416

UNDER-EXPRESSED	GO:0016071	mRNA metabolic process	-0.20209	-2.0828	0.021411	0.421
UNDER-EXPRESSED	GO:0006260	DNA replication	-0.30328	-2.07723	0.021637	0.433
UNDER-EXPRESSED	GO:0000278	mitotic cell cycle	-0.35951	-2.00917	0.03053	0.576
UNDER-EXPRESSED	GO:0022618	ribonucleoprotein complex assembly	-0.33305	-2.00875	0.029781	0.576
UNDER-EXPRESSED	GO:0051248	negative regulation of protein metabolic process	-0.43041	-1.99818	0.030706	0.612
UNDER-EXPRESSED	GO:0032269	negative regulation of cellular protein metabolic process	-0.43041	-1.96581	0.035649	0.675
UNDER-EXPRESSED	GO:0071826	ribonucleoprotein complex subunit organization	-0.33305	-1.95843	0.036662	0.693
UNDER-EXPRESSED	GO:0000375	RNA splicing, via transesterification reactions	-0.24297	-1.93533	0.038547	0.739
UNDER-EXPRESSED	GO:0044092	negative regulation of molecular function	-0.37487	-1.91118	0.044203	0.786
UNDER-EXPRESSED	GO:0030163	protein catabolic process	-0.23014	-1.90654	0.044658	0.797
UNDER-EXPRESSED	GO:0043632	modification-dependent macromolecule catabolic process	-0.25771	-1.89874	0.045812	0.813

Supplementary Table S2. Gene Set Enrichment Analysis- Cellular Component GO terms over- and under-expressed in the aesthetasc-bearing region of the antennules in adult *Panulirus ornatus*.

Tags	GO ID	GO Name	ES	NES	FDR q-val	FWER p-val
OVER-EXPRESSED	GO:0005794	Golgi apparatus	0.269095	2.346675	0.005897	0.091
OVER-EXPRESSED	GO:0031984	organelle subcompartment	0.244055	2.326708	0.006029	0.097
OVER-EXPRESSED	GO:0070469	respirasome	0.41968	2.18709	0.014457	0.238
OVER-EXPRESSED	GO:0098791	Golgi apparatus subcompartment	0.37081	1.981138	0.042134	0.638
UNDER-EXPRESSED	GO:0000785	chromatin	-0.27826	-1.88302	0.045885	0.835
UNDER-EXPRESSED	GO:1990234	transferase complex	-0.19455	-1.87277	0.046888	0.851
UNDER-EXPRESSED	GO:0005615	extracellular space	-0.33755	-1.85518	0.049363	0.868
UNDER-EXPRESSED	GO:0005694	chromosome	-0.3414	-3.34063	0	0
UNDER-EXPRESSED	GO:0022626	cytosolic ribosome	-0.5443	-2.77939	9.20E-04	0.004
UNDER-EXPRESSED	GO:0005730	nucleolus	-0.44475	-2.67829	0.001351	0.007
UNDER-EXPRESSED	GO:0044391	ribosomal subunit	-0.33109	-2.45441	0.005994	0.05
UNDER-EXPRESSED	GO:0099080	supramolecular complex	-0.28051	-2.33178	0.007402	0.102
UNDER-EXPRESSED	GO:0005874	microtubule	-0.33915	-2.28863	0.009086	0.14
UNDER-EXPRESSED	GO:0099513	polymeric cytoskeletal fiber	-0.32118	-2.26127	0.010438	0.167
UNDER-EXPRESSED	GO:0099081	supramolecular polymer	-0.3051	-2.24536	0.010772	0.179
UNDER-EXPRESSED	GO:0099512	supramolecular fiber	-0.3051	-2.19624	0.013491	0.232
UNDER-EXPRESSED	GO:0005654	nucleoplasm	-0.24556	-2.1208	0.01774	0.337
UNDER-EXPRESSED	GO:0015935	small ribosomal subunit	-0.38614	-2.00631	0.029478	0.583
UNDER-EXPRESSED	GO:0015934	large ribosomal subunit	-0.35051	-1.94255	0.037889	0.725

Supplementary Table S3. Gene Set Enrichment Analysis- Molecular Function GO terms over- and under-expressed in the aesthetasc-bearing region of the antennules in adult *Panulirus ornatus*.

Tags	GO ID	GO Name	ES	NES	FDR q-val	FWER p-val
OVER-EXPRESSED	GO:0004970	ionotropic glutamate receptor activity	0.608033	2.914796	0	0
OVER-EXPRESSED	GO:0009055	electron transfer activity	0.402743	2.897757	0	0
OVER-EXPRESSED	GO:0008066	glutamate receptor activity	0.608033	2.865916	0	0
OVER-EXPRESSED	GO:0030594	neurotransmitter receptor activity	0.560229	2.847563	0	0
OVER-EXPRESSED	GO:0022824	transmitter-gated ion channel activity	0.560229	2.829917	0	0
OVER-EXPRESSED	GO:0022835	transmitter-gated channel activity	0.560229	2.805225	0	0
OVER-EXPRESSED	GO:0005230	extracellular ligand-gated ion channel activity	0.421803	2.51709	0.001789	0.024
UNDER-EXPRESSED	GO:0061134	peptidase regulator activity	-0.32261	-1.85483	0.048597	0.868
UNDER-EXPRESSED	GO:0030414	peptidase inhibitor activity	-0.32261	-1.85138	0.048769	0.871
UNDER-EXPRESSED	GO:0005543	phospholipid binding	-0.39706	-1.84485	0.049891	0.879
UNDER-EXPRESSED	GO:0005102	signaling receptor binding	-0.45818	-2.4595	0.006461	0.049
UNDER-EXPRESSED	GO:0005200	structural constituent of cytoskeleton	-0.40952	-2.34864	0.007401	0.093
UNDER-EXPRESSED	GO:0016810	hydrolase activity, acting on carbon-nitrogen (but not peptide) bonds	-0.43392	-2.31155	0.00812	0.116
UNDER-EXPRESSED	GO:0016410	N-acyltransferase activity	-0.42145	-2.04772	0.025174	0.501
UNDER-EXPRESSED	GO:0008080	N-acetyltransferase activity	-0.41726	-1.98665	0.032283	0.641
UNDER-EXPRESSED	GO:0016407	acetyltransferase activity	-0.40674	-1.95812	0.035847	0.693
