# Supplementary figures and images for: Tropical rock lobster (Panulirus ornatus) uses chemoreception via the antennular lateral flagellum to identify conspecific ecdysis
Source: Sci Rep. 2023 Jul 31;13:12409. doi: 10.1038/s41598-023-39567-8 (PMC10390513; doi:10.1038/s41598-023-39567-8)

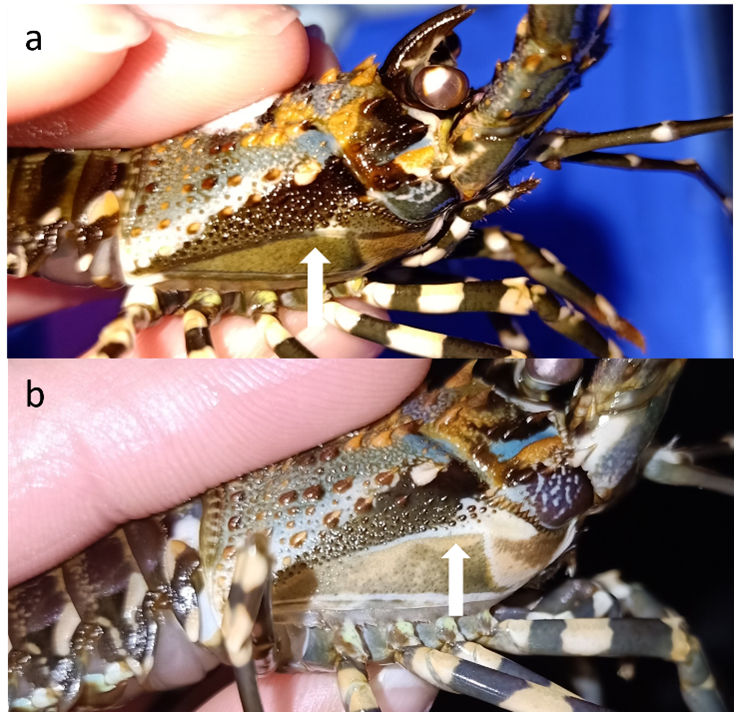

Supplement: Supplementary file 2 — Supplementary Figures. [file 41598_2023_39567_MOESM2_ESM.png]
